# Preliminary transcriptome profiling of induced pluripotent stem cell-derived hematopoietic stem and progenitor cells in Kawasaki disease

**DOI:** 10.3389/fped.2026.1833402

**Published:** 2026-08-19

**Authors:** Lianni Mei, Lei Gao, Ruizhi Zhang, Qingzhu Qiu, Qiuping Lin, Libing Shen, Wenyuan Wang, Lijian Xie

**Affiliations:** 1 Department of Pediatrics, Jinshan Hospital, Fudan University, Shanghai, China; 2 Department of Cardiology, Shanghai Children’s Hospital, School of Medicine, Shanghai Jiao Tong University, Shanghai, China; 3 Longhua Hospital, Shanghai University of Traditional Chinese Medicine, Shanghai, China; 4 Interdisciplinary Research Center on Biology and Chemistry, Shanghai Institute of Organic Chemistry, Chinese Academy of Sciences, Shanghai, China; 5 Institute of Pediatric Infection, Immunity, and Critical Care Medicine, School of Medicine, Shanghai Jiao Tong University, Shanghai, China

**Keywords:** bulk RNA-seq, hematopoietic stem and progenitor cells, induced pluripotent stem cells, Kawasaki disease, scRNA-seq

## Abstract

**Background:**

The etiology of Kawasaki disease (KD) remains unclear. Hematopoietic stem and progenitor cells (HSPCs) serve as the precursor cells for a multitude of immune cells. Investigating their initial transcriptional status may help uncover the aberrant immune mechanisms underlying KD.

**Methods:**

Induced pluripotent stem cells (iPSCs) were reprogrammed from peripheral blood mononuclear cells (PBMCs) isolated from patients with KD and febrile individuals, followed by directed differentiation into HSPCs. We performed bulk RNA-sequencing to compare transcriptomic profiles of iPSC-derived HSPCs between the two groups, with further comparison against integrated HSPC data from public KD single-cell datasets.

**Results:**

We recruited three patients with KD prior to Intravenous immunoglobulin (IVIG) therapy and three febrile control patients, and successfully established an iPSC-derived HSPCs disease model. Transcriptomic profiling revealed elevated immune and inflammatory response signatures in iPSC-derived HSPCs from patients with KD compared with those from febrile individuals. In addition, *in vitro*-generated KD iPSC-HSPCs exhibited partial transcriptional features similar to *in vivo* HSPCs isolated from PBMCs of patients with KD. Gene Set Enrichment Analysis (GSEA) further revealed that gene sets associated with B-cell developmental processes were transcriptionally downregulated in iPSC-derived HSPCs from patients with KD relative to febrile controls.

**Conclusions:**

iPSC-derived HSPCs from patients with KD display altered immune-inflammatory transcriptional profiles and suppressed B-cell developmental signatures at the transcriptomic level. These preliminary findings suggest that early hematopoietic immune dysregulation may contribute to KD pathogenesis. We propose that these iPSC-derived HSPCs could be a good cellular model for studying the etiology of KD *in vitro*. These findings are preliminary, constrained by the small sample size and limited to transcriptomic analysis only. Further studies with larger cohorts and functional experiments are needed to verify these results.

## 1 Introduction

Kawasaki disease (KD) is an acute systemic vasculitis that primarily occurs in young children and represents the leading cause of acquired heart disease in pediatric populations worldwide (1). Coronary artery lesions (CALs) represent its major complication. Despite decades of extensive research, the exact etiology and pathogenic mechanisms of KD remain unclear. Three prevailing hypotheses, including superantigen activation (2), RNA viral infection (3), and genetic susceptibility (4), fail to fully explain the etiology and complex pathogenesis of KD. It is most widely accepted that KD is driven by excessive immune responses to environmental and infectious triggers in genetically susceptible children (5, 6). High-resolution single-cell transcriptomics provides an opportunity to characterize such immune abnormalities. Recent single-cell RNA sequencing (scRNA-seq) studies of peripheral blood mononuclear cells (PBMCs) from patients with KD have deepened our understanding of immune dysregulation in KD. Existing single-cell evidence has documented robust inflammatory activation of monocytes, substantial phenotypic and functional perturbations in T-cell subsets, and impaired developmental and transcriptional signatures of B cells during acute KD (7–9). Nevertheless, these PBMC-derived observations only depict aberrant immune phenotypes of mature circulating immune cells. During the acute KD phase, dynamic perturbations of immune cell populations occur, with hematopoietic stem and progenitor cells (HSPCs) emerging as a key research target owing to their capacity to give rise to multiple immune-cell lineages. One prior study specifically compared the expression profiles of HSPCs between patients with KD prior to Intravenous immunoglobulin (IVIG) therapy and febrile individuals (10). The analysis identified activated IL-17 signaling and upregulated biological processes related to immune response and inflammatory response, suggesting an inherent predisposition toward premature immune activation in patients with KD. However, direct *in vitro* cellular evidence remains insufficient.

**Figure 1:**
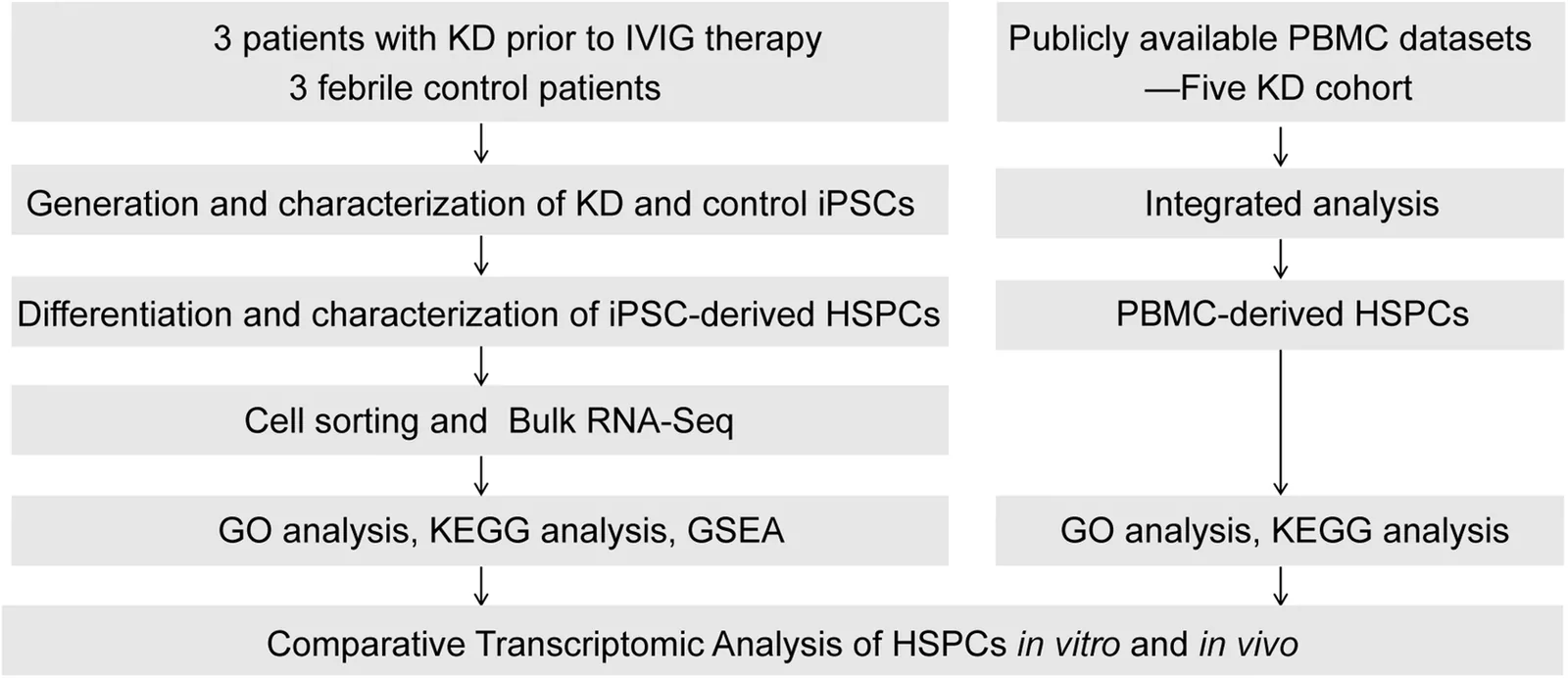
The study flow chart. KD, Kawasaki disease; IVIG, intravenous immunoglobulin; iPSCs, induced pluripotent stem cells; HSPCs, hematopoietic stem and progenitor cells; GO analysis, gene ontology analysis; KEGG analysis, kyoto encyclopedia of genes and genomes analysis; GSEA, gene set enrichment analysis; PBMC, peripheral blood mononuclear cell.

Another pioneering study established an induced pluripotent stem cell (iPSC)-derived vascular endothelial cell model for KD, identifying elevated *CXCL12* as a potential driver of IVIG resistance and validating the utility of iPSC-based cellular model for investigating KD pathogenesis (11). Since human iPSCs can be differentiated into functional HSPCs (12), this strategy facilitates investigation of immune dysregulation in KD. In this study, we established an *in vitro* iPSC-derived HSPC model, performed transcriptomic profiling of HSPCs from patients with KD in the acute phase and febrile controls, and conducted integrated bioinformatic analyses to identify key signaling pathways and molecules underlying KD-associated immune abnormalities.

## 2 Materials and methods

### 2.1 Participants

This study enrolled patients with KD and febrile individuals at the Department of Pediatrics, Jinshan Hospital, Fudan University. The study was reviewed and approved by the Ethics Committee of Jinshan Hospital (IRB No. 2024-S101). Written informed consent was obtained from the participants' legal guardians/next of kin for the publication of any potentially identifiable images or data included in this article. The inclusion and exclusion criteria for patients were consistent with those reported in a previously published article (10). Blood samples collected from patients with KD prior to IVIG therapy and from febrile patients immediately upon admission. PBMCs were isolated via density gradient centrifugation using Ficoll-Paque Premium.

### 2.2 Generation of iPSCs

iPSCs were generated from PBMCs of all patients using the CytoTune iPS 2.0 Sendai Reprogramming Kit carrying OCT4, SOX2, KLF4, and c-Myc (ThermoFisher, A16517), as previously described (13, 14). Briefly, approximately 1.5 × 10^5^ PBMCs were seeded into 24-well plates and cultured in Stempro34 medium supplemented with stem cell factor, FMS-like tyrosine kinase 3 ligand, interleukin-3, and interleukin-6. Four days later, the cells were transduced with the Sendai virus-based reprogramming vectors. Twenty-four hours post-transduction, the somatic cell culture medium was refreshed daily. On day 3 after transduction, cells were counted, and 5,000 viable cells were replated into 12-well plates for continued culture. Subsequently, the culture medium was replaced with Stemflex medium every other day to support iPSC colony formation. Approximately 3–4 weeks after viral transduction, distinct single-cell iPSC colonies were manually isolated, then subjected to further expansion and characterization.

### 2.3 Karyotype analysis

G-banded karyotyping was conducted by Zhen He Biotechnology (Shanghai, China) at the 15th cell passage. Cells were exposed to 0.1 mg/mL colcemid to induce mitotic arrest, then collected via accutase dissociation, hypotonic treatment, and fixation in a 3:1 methanol-acetic acid solution. Twenty metaphase spreads were examined for each cell line.

### 2.4 Immunofluorescence assay

The iPSCs (passage 15) were cultured on coverslips and fixed with 4% paraformaldehyde for 30 min at room temperature. Then, the cells were washed with 1 × Phosphate-Buffered Saline (PBS) and incubated in 1 × PBS with 10% normal goat serum and 0.3% Triton X-100 for 30 min at room temperature. Primary antibodies against NANOG, SOX2, and OCT4 (Abcam and Thermo Fisher Scientific) were diluted 1:200 in blocking solution, while the primary antibody against SSEA4 (Abcam) was diluted 1:400 in blocking solution. All antibodies were incubated with cells overnight at 4 °C. After washing with 1 × PBS three times, the secondary antibodies (Thermo Fisher Scientific) were diluted in blocking solutions (1:200) and incubated with cells for 1 h at room temperature. The cells were then washed three times with 1 × PBS. The nuclei were counterstained with DAPI. Following three washes with 1 × PBS, the coverslips were mounted on slides using an anti-fade mounting solution. Images were taken with Leica SP8 confocal microscope. Image J was used for image analysis.

### 2.5 Differentiation of iPSCs into HSPCs

Differentiation of iPSCs into HSPCs was performed by STEMdiff™ Hematopoietic Kit (15) (Stemcell, 05310). On Day −1, harvest and seed iPSCs as small aggregates in a 6-well plate. then culture the cells with Stemflex medium. On Day 0 (once verifying that adhered colonies are present at a density of 4–10 per cm^2^), replace Stemflex medium with Medium A to direct cells toward a mesoderm-like lineage. On Day 2, perform a half-volume medium refreshment using fresh Medium A. On Day 3, switch to Medium B and conduct half-medium changes on Days 5, 7, and 10 to support further differentiation toward hematopoietic cells. Generally, abundant HSPCs can be collected from the culture supernatant by Day 12.

### 2.6 Flow cytometry and cell sorting

On day 12 of culture, suspended cells were harvested and washed twice with Stain Buffer (BD Pharmingen, 554656). Subsequently, the cells were incubated with Fc blocking reagent (STARTER, S0F0002) for 10 min. According to the manufacturer's instructions, Fixable Viability Stain 510 (BD Pharmingen, 564406) was added to cell suspensions to distinguish viable and dead cells, followed by 15 min of incubation at room temperature in the dark. Then, CD34 (BD Pharmingen, 550761), CD43 (BD Pharmingen, 562916) and CD45 (BD Pharmingen, 555482) antibodies were added as instructed, and cells were incubated on ice for 20 min away from light. Flow cytometric data were acquired using a BD LSRFortessa flow cytometer, with a minimum of 10,000 events collected for each sample. Data analysis was performed using FlowJo V10 software. For cell sorting, cells were pretreated following the identical staining procedures described above except for the conjugated antibodies used. Briefly, cells were stained with CD235a (BD Biosciences, Cat: 757574) and CD43 (BD Biosciences, Cat: 562916). Flow cytometric analysis and cell sorting were subsequently conducted on a BD Influx cell sorter. Finally, sorted target cells were collected into centrifuge tubes prefilled with fetal bovine serum (FBS) for subsequent experiment.

### 2.7 Bulk RNA-sequencing

HSPCs were first isolated through sorting and subsequently rinsed with PBS. Total RNA isolation was carried out employing the MJzol Animal RNA Isolation Kit (Majorivd), strictly following the manufacturer's established protocols. To eliminate genomic DNA contamination, the RNA was further purified utilizing both the RNAClean XP Kit (Beckman Coulter) and the RNase-Free DNase Set (Qiagen). The purity and concentration of the harvested total RNA were assessed by measuring the absorbance ratio at 260/280 nm using a UV spectrophotometer. For transcriptome analysis, library preparation and sequencing were performed on the Illumina NovaSeq 6,000 platform, employing a paired-end 150 bp (PE150) strategy. The resulting sequence reads were mapped to the human reference genome (hg38) using Hisat2. Subsequently, StringTie was applied to the aligned data to generate quantitative gene expression profiles. Finally, expression levels for each gene were quantified in RPKM and subsequently transformed to a log2(RPKM + 1) scale for downstream analysis.

### 2.8 RNA-seq data analysis

To identify differentially expressed genes (DEGs) between the predefined groups, the R package DEGseq was applied, using a fold change threshold of ≥1. A principal component analysis (PCA) was performed, incorporating the 5,000 transcripts with the highest expression levels. Functional enrichment analysis was performed using the DAVID Functional Annotation Bioinformatics Microarray Analysis online tool (https://davidbioinformatics.nih.gov/home.jsp). Gene Ontology (GO) term and Kyoto Encyclopedia of Genes and Genomes (KEGG) pathway with an FDR value below 0.05 were considered statistically meaningful and kept for further analysis. Gene Set Enrichment Analysis (GSEA) was executed using the GSEA v4.4.0 tool, strictly following the official guidelines. The analysis utilized several annotated gene sets from MSigDB v2025: the c2.all.v2025.symbols.gmt collection for KEGG pathway annotations, the c7.all.v2025.symbols.gmt collection for immunological signatures, and the c8.all.v2025.symbols.gmt collection for cell type signatures. The specific parameters configured for GSEA included 1,000 permutations, a gene_set permutation type, a weighted enrichment statistic, and the Diff_of_Classes metric for ranking genes, with a maximum gene set size of 6,000 and a minimum size of 1. B cell signature genes were extracted from RNA-Seq data of two groups, and then plotted using boxplots in R language.

### 2.9 scRNA-seq data analysis

The scRNA-seq datasets analyzed in this study were obtained from the The National Center for Biotechnology Information Gene Expression Omnibus and China National Center for Bioinformation repositories, under the accession codes GSE168732, GSE200743, GSE183716, GSEqiuqiu, and OMIX007142. Initial alignment of sequencing reads to the human reference genome (GRCh38) was carried out using Cell Ranger. Subsequent data processing and filtering were performed with the Seurat package (version 4.0.2). To maintain data quality, we applied thresholds requiring unique molecular identifier (UMI) counts to range between 200 and 6,000, while also limiting the proportions of mitochondrial, hemoglobin, and ribosomal genes to below 10%, 0.1%, and 3%, respectively. To address over-dispersion and facilitate dataset integration, normalization was conducted using the SCTransform method. This normalization was first applied independently to each of the five individual datasets. For integration, 2,000 common features were selected across all samples, and the FindIntegrationAnchors function was employed to combine them. Following integration, a second SCTransform was performed to generate a unified, normalized matrix. The resulting integrated data were then scaled, and PCA was conducted. The top principal components were subsequently used as input for two-dimensional visualization via the uniform manifold approximation and projection (UMAP) algorithm. To identify distinct cell populations within the PBMCs, clustering was performed on the PCA-reduced data based on shared nearest neighbor graph construction. Refinement of cluster annotations was achieved by examining the expression of canonical marker genes, with reference to the CellMarker 2.0 database (http://117.50.127.228/CellMarker/). Final assignment of cell identities was further corroborated through multimodal reference mapping utilizing the SeuratDisk package.

## 3 Results

### 3.1 Clinical features and immunological parameters of KD and febrile

In this study, we recruited 3 patients with KD prior to IVIG therapy and 3 febrile control patients. The patients with KD (1 male, 2 females; aged 1.9–2.7 years, <5 years) were diagnosed with complete KD, showed IVIG sensitivity, and did not develop CALs. The febrile patients (2 male, 1 female; aged 2.0–2.8 years, <5 years) presented with pneumonia (Table 1). A comparative analysis of immune cell proportions and immunoglobulin levels was conducted between the two groups. The percentage of CD4+ T cells in the KD group (33.97 ± 6.13%) was lower than that in the control group (47.82 ± 2.69%, *P* < 0.05). Similarly, the level of immunoglobulin A (IgA) in the KD group (0.54 ± 0.24) was also reduced compared with the control group (1.25 ± 0.32, *P* < 0.05). The CD19+ cell proportion in the KD group showed a decreasing trend (16.60 ± 3.94% vs. 22.70 ± 5.46% in the control group) without statistical significance. No significant differences were observed in other indicators (Table 2).

**Table 1:** Clinical features of patients with KD and febrile controls.

Features	KD 1	KD 2	KD 3	Febrile 1	Febrile 2	Febrile 3
Age (Years)	2.4	1.9	2.7	2.8	2.0	2.1
Sex (male/female)	Male	Female	Female	Male	Male	Female
Fever (Days)	7	5	5	5	7	5
Diagnosis	Complete KD	Complete KD	Complete KD	Pneumonia	Pneumonia	Pneumonia
IVIG sensitive	Yes	Yes	Yes	NA	NA	NA
CALs	No	No	No	NA	NA	NA

KD, Kawasaki disease; IVIG, intravenous immunoglobulin; CALs, coronary artery lesions.

**Table 2:** Immunological parameters of patients with KD and febrile controls.

Immunological parameters	KD (*n* = 3)	Febrile (*n* = 3)
CD19+ (%)	16.60 ± 3.94	22.70 ± 5.46
CD3+ (%)	70.51 ± 2.57	70.40 ± 4.47
CD4+ (%)	33.97 ± 6.13*	47.82 ± 2.69*
CD8+ (%)	27.62 ± 8.97	17.78 ± 7.40
CD4+/CD8+ (%)	1.36 ± 0.62	3.11 ± 1.56
CD16 + 56+/CD3− (%)	11.16 ± 4.91	5.74 ± 3.75
IgG (g/L)	9.07 ± 4.78	10.09 ± 4.36
IgA (g/L)	0.54 ± 0.24*	1.25 ± 0.32*
IgM (g/L)	1.01 ± 0.35	1.20 ± 0.51
IgE (IU/mL)	96.33 ± 38.79	61.67 ± 60.05

KD, Kawasaki disease; IgG, immunoglobulin G; IgA, immunoglobulin A; IgM, immunoglobulin M; IgE, immunoglobulin E.

* A statistically significant difference (*P* < 0.05).

### 3.2 Construction of an iPSC-derived HSPCs model for KD and febrile

**Figure 2:**
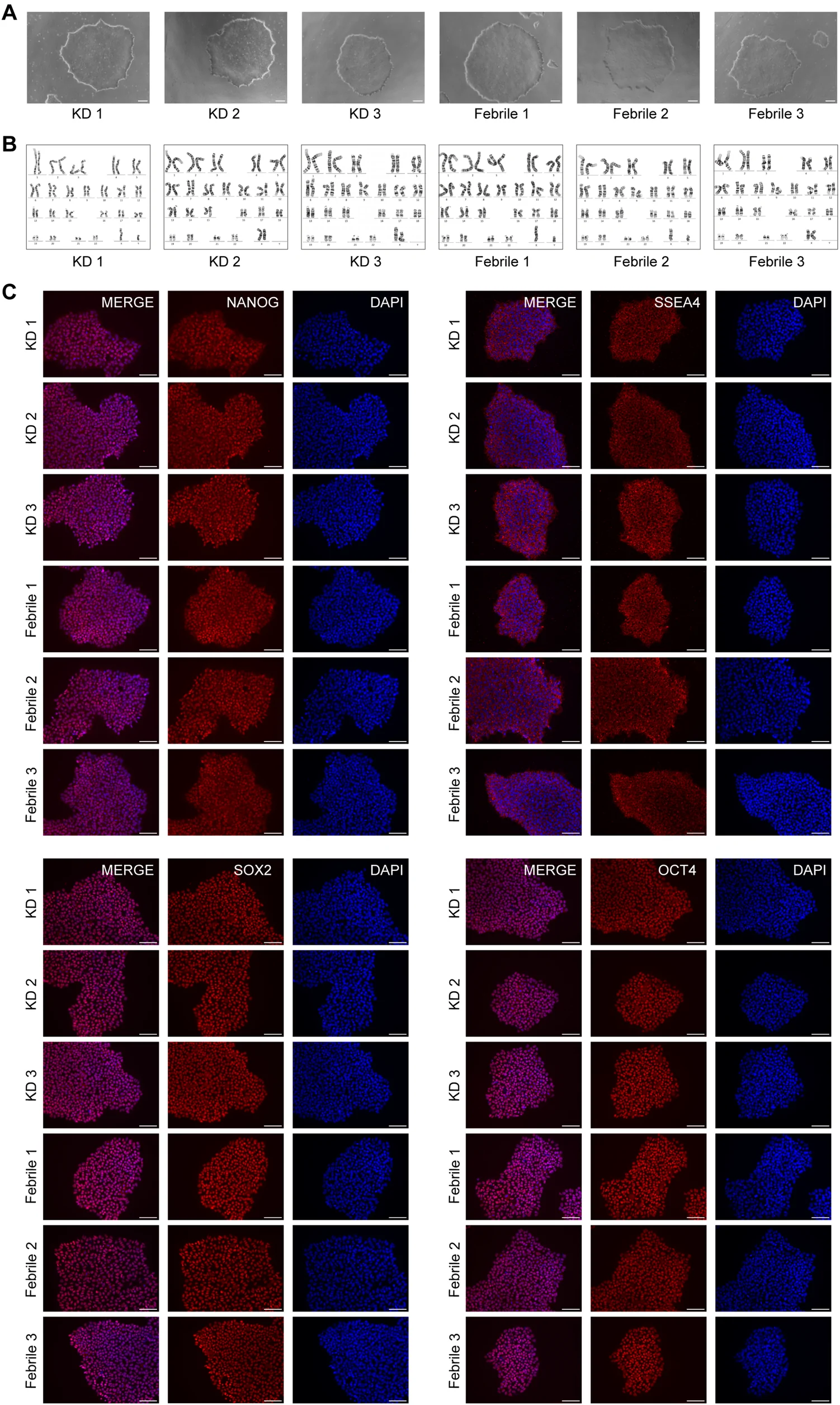
Generation and characterization of patient-specific Kawasaki disease (KD) and control induced pluripotent stem cells (iPSCs). Schematic scheme of the iPSC colonies, showing the typical morphology of human embryonic stem cells. Scale bars, 200 µm. **(A)** Three KD-iPSCs and three febrile-iPSCs had normal karyotypes. **(B)** Schematic scheme of iPSC colonies immunofluorescence with markers for the undifferentiated state (NANOG, SSEA4, SOX2, and OCT4). Nuclei were stained with DAPI. Scale bars, 100 µm.

**Figure 3:**
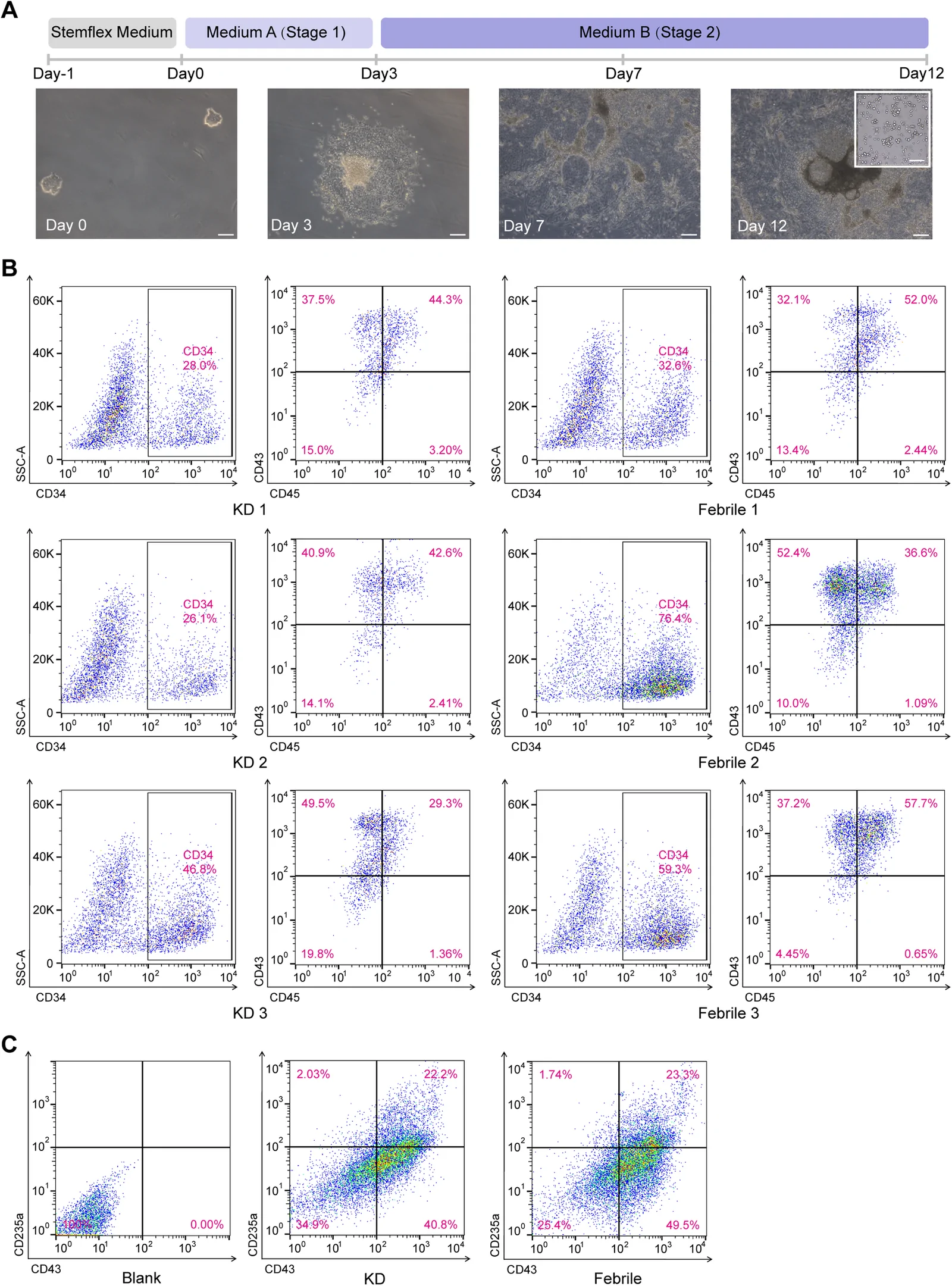
Differentiation of iPSCs into hematopoietic stem and progenitor cells (HSPCs) and flow-cytometric assessment and sorting of iPSC-derived HSPCs. **(A)** Schematic scheme of the direct differentiation from iPSCs to HSPCs. Scale bars, 200 µm. **(B)** Flow Cytometry: Gating strategy and flow cytometric characterization of KD and febrile iPSC-derived HSPCs for CD34, CD43 and CD45 expression at day 12. **(C)** Cell Sorting: Representative dot plots of KD and febrile iPSC-derived HSPCs for CD235a and CD43 expression at day 12 of differentiation.

PBMCs were obtained from the recruited six patients. iPSCs were then generated from PBMCs by using Sendai-viral transduction. The generated iPSC lines exhibited typical human embryonic stem cell-like morphology when cultured in Stemflex medium (Figure 2A). Karyotype analysis via G-banding confirmed normal chromosomal number and structure (Figure 2B). Pluripotency was validated by immunofluorescence staining of stem cell markers (NANOG, SSEA4, SOX2, and OCT4; Figure 2C), demonstrating successful somatic cell reprogramming. Differentiation into HSPCs was performed using the STEMdiff™ Hematopoietic Kit. iPSCs were cultured on Matrigel without feeder cells, induced toward mesoderm with medium A for 3 days, then transitioned to medium B for 9 days (Figure 3A). Suspended cells were harvested from differentiation cultures and analyzed for immunophenotype with hematopoietic markers including CD34, CD43 and CD45 (Figure 3B). HSPCs were sorted using the CD235a-CD43+ marker panel (Figure 3C), a unbiased sorting approach described in a previous study (16). Collectively, these results support the successful establishment of an iPSC-derived HSPCs model for studying KD.

### 3.3 Elevated immune and inflammatory responses observed in iPSC-derived HSPCs from patients with KD

**Figure 4:**
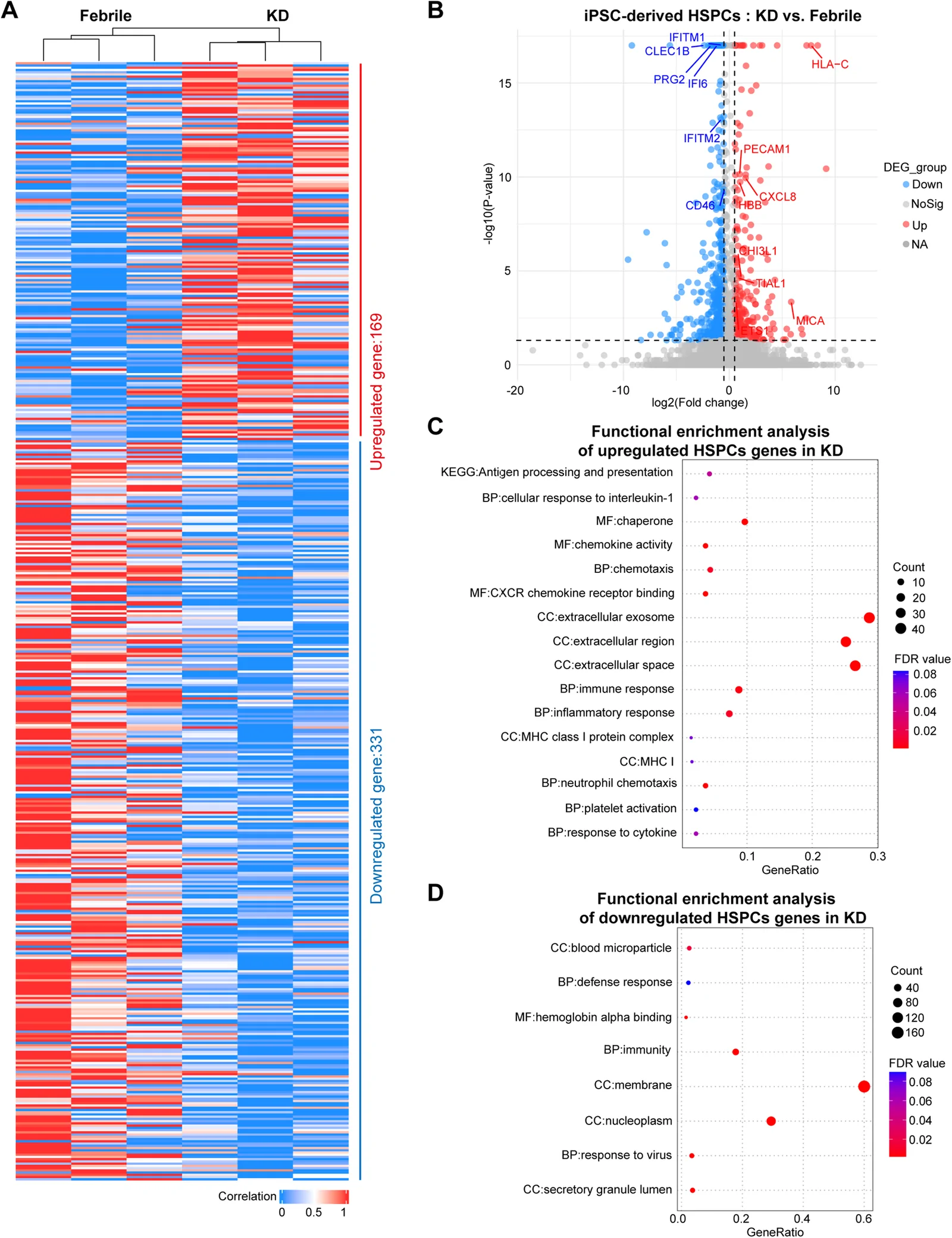
Transcriptomic profiling and functional enrichment analysis of iPSC-derived HSPCs between KD and febrile. **(A)** Heatmap based on the differentially expressed genes (DEGs) between KD and febrile. **(B)** Volcano plot of DEGs between KD and febrile. Cutoff for log2(fold change) was set to 1 and −1, and *P* < 0.05. **(C)** Functional enrichment analysis of upregulated DEGs in KD. **(D)** Functional enrichment analysis of downregulated DEGs in KD. For 4C to 4D, BP, Biological Process; CC, Cellular Component; MF, Molecular Function; KEGG, KEGG Pathway.

Using RNA sequencing, we analyzed the expression profiles of iPSC-derived HSPCs from patients with KD before IVIG treatment and febrile controls. PCA showed a separation between the two groups, with each displaying distinctive gene expression signatures (Supplementary Figure S1A). Differential gene expression analysis identified 169 upregulated and 331 downregulated genes in the KD compared to febrile groups (Figures 4A,B). Functional enrichment analysis of these DEGs further revealed distinct immune-related biological patterns (Figures 4C,D). Upregulated genes in KD-iPSC derived HSPCs were predominantly associated with immune response, inflammatory response, and neutrophil chemotaxis, with enrichment in extracellular exosome, extracellular region, and extracellular space. In contrast, downregulated genes were primarily involved in immunity, defense response, and response to virus, with localization in the membrane, nucleoplasm, and secretory granule lumen. These findings suggest patients with KD rely on distinct immune mechanisms compared to febrile controls, characterized by enhanced immune and inflammatory responses in iPSC-derived HSPCs, which may further indicate premature immune activation in KD.

### 3.4 Comparative transcriptomic analysis of HSPC in KD

**Figure 5:**
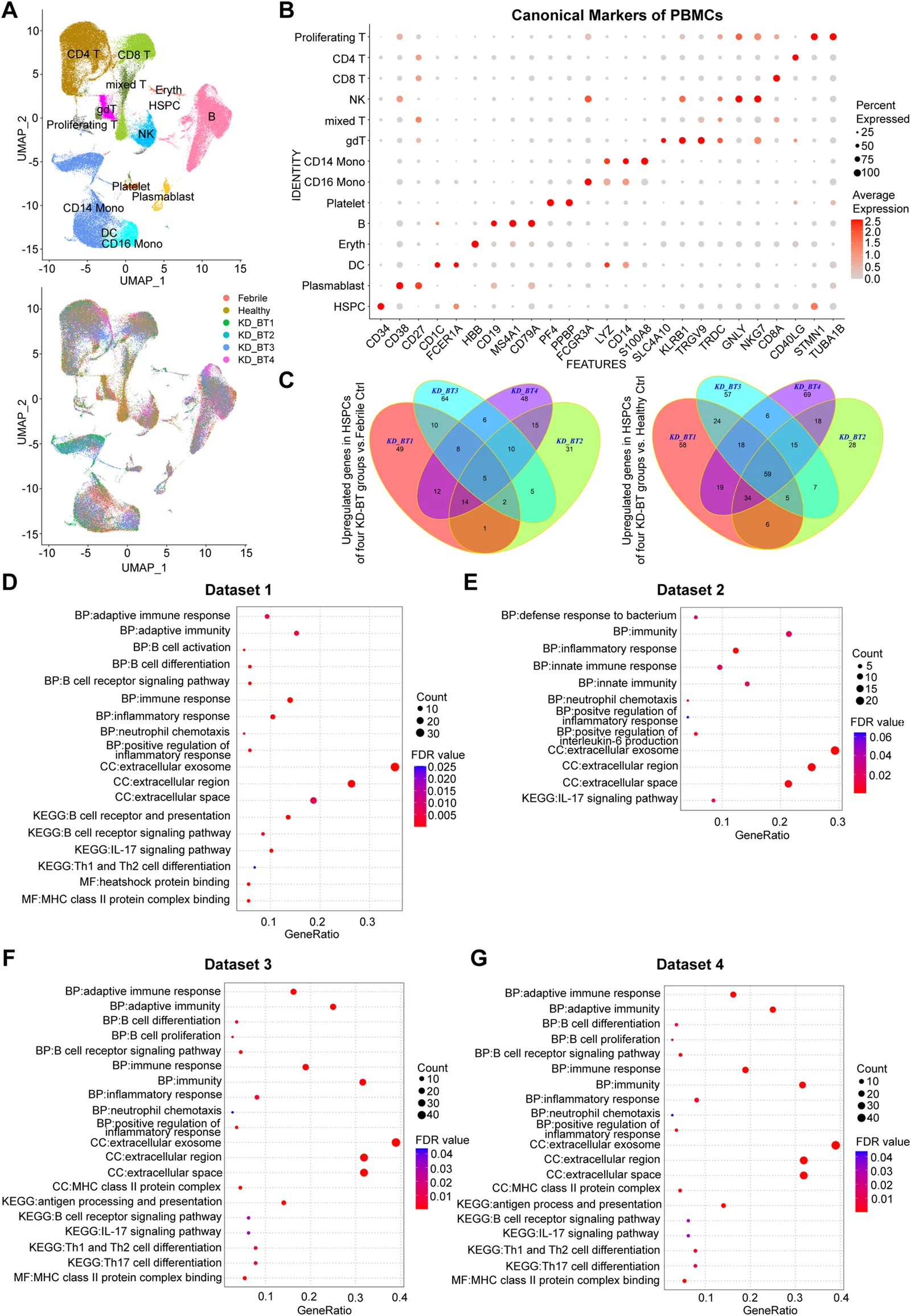
HSPC subset extraction and functional enrichment of upregulated DEGs across five KD single-cell datasets. **(A)** The integration of single-cell profiling analysis of KD datasets, which include 15 patients with KD before IVIG treatment (KD_BT), three febrile and three healthy patients as two control groups. **(B)** Expression of canonical cell-type-specific gene markers in KD, febrile, and healthy individuals based on integrated analysis. **(C)** Venn diagram of upregulated genes for patients with KD in four groups (febrile patients and healthy patients as the control group). **(D)** Functional enrichment analysis of upregulated DEGs in KD dataset 1. **(E)** Functional enrichment analysis of upregulated DEGs in KD dataset 2. **(F)** Functional enrichment analysis of upregulated DEGs in KD dataset 3. **(G)** Functional enrichment analysis of upregulated DEGs in KD dataset 4. For 5D to 5G, BP, biological process; CC, cellular component; MF, molecular function; KEGG, KEGG pathway.

To investigate the transcriptional similarities between HSPCs derived from iPSCs (*in vitro*) and those from human PBMCs (*in vivo*), we conducted an integrated analysis of publicly available PBMC datasets. After stringent quality control, the final dataset comprised 102,398 cells across five KD cohorts: 16,000 cells (KD_BT_GSE168732), 12,000 cells (KD_BT_GSE200743), 15,400 cells (KD_BT_GSE183716), and 13,000 cells (KD_BT_GSEqiuqiu), alongside 23,600 cells from febrile controls (Febrile_Ctrl_GSEqiuqiu) and 22,398 cells from healthy controls (Healthy_Ctrl_ OMIX007142). Unsupervised clustering identified 14 major cell types (Figure 5A, Supplementary Figure S1C), with HSPCs marked by CD34 expression constituting a minor population (Figure 5B). Single-cell transcriptome analysis demonstrated substantial heterogeneity among HSPCs of patients with KD, with only 5 common DEGs identified across all four datasets when compared to febrile controls, but 59 common DEGs identified across all four datasets when compared to healthy controls (Figure 5C). PCA revealed that compared with healthy controls, patients with KD before IVIG treatment (KD_BT) and febrile controls were more distinctly separated from each other (Supplementary Figure S1B). Subsequently, we additionally compared the expression profiles of HSPCs between KD_BT and febrile control. Comparative analysis of upregulated genes revealed that iPSC-derived HSPCs and PBMC-derived HSPCs of patients with KD shared some common transcriptional features, particularly enrichment in biological processes including immune response, inflammatory response, and neutrophil chemotaxis (Figures 5D–G). Notably, these cell types also exhibited shared cellular localizations (extracellular exosome, extracellular region, and extracellular space).

### 3.5 KD iPSC-derived HSPCs display transcriptional signatures consistent with impaired B-cell developmental programs

**Figure 6:**
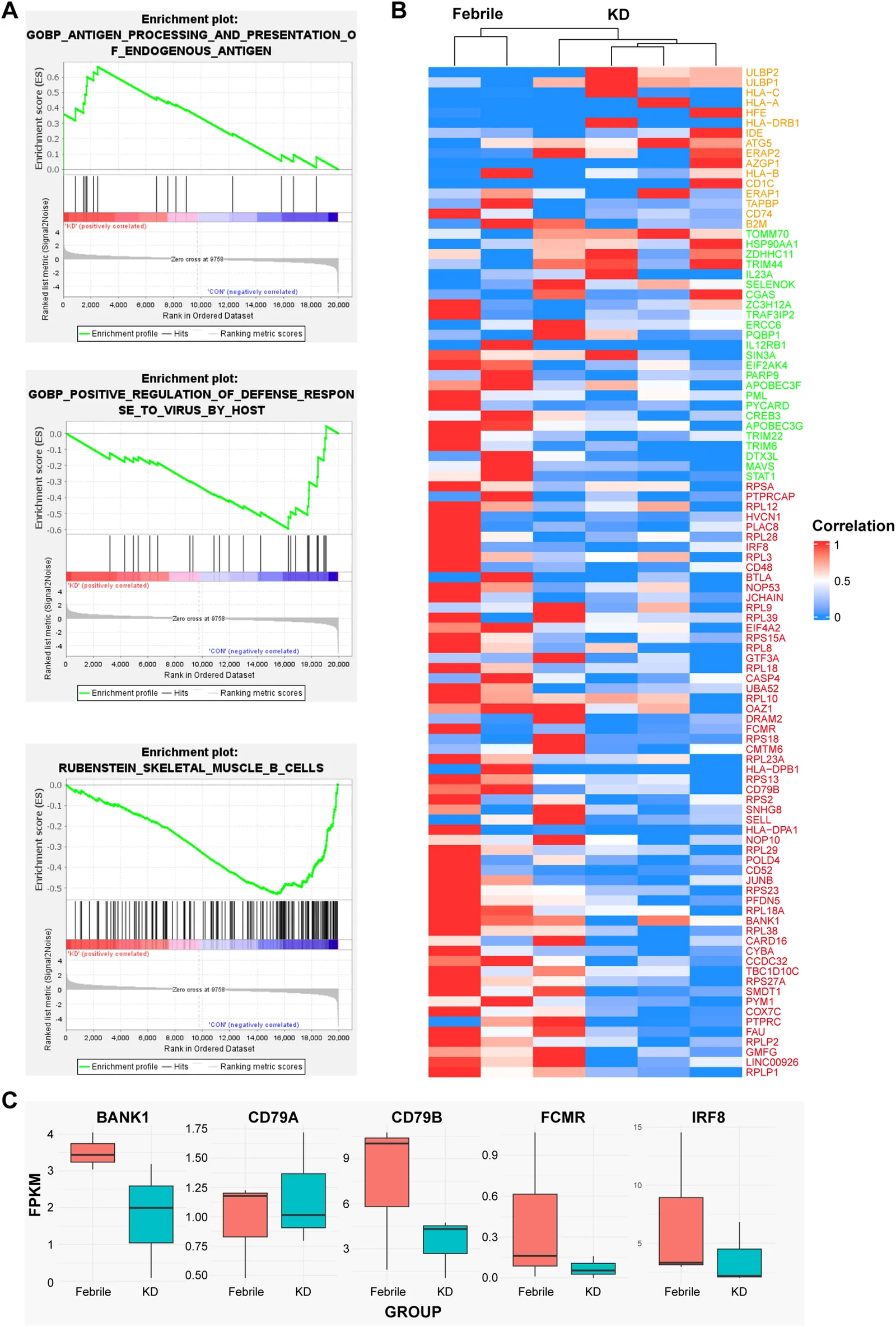
Gene set enrichment analysis (GSEA) and expression analysis of B-cell signature genes in iPSC-derived HSPCs from KD and febrile groups. **(A)** GSEA plots show the downregulation of B-cell genes, downregulation of positive regulation of defense response to virus by host genes and upregulation of antigen processing and presentation of endogenous antigen genes in KD iPSC-derived HSPCs relative to febrile iPSC-derived HSPCs. **(B)** The hierarchical clustering heatmap of GSEA-enriched genes in **(A) (C)** Expression comparison between two group for each B cell signature gene.

We performed GSEA using RNA-seq data from iPSC-derived HSPCs of KD and febrile groups. The results showed significant downregulation of B-cell-related gene sets in KD iPSC-derived HSPCs (Figure 6A). Concurrently, gene sets associated with antigen processing and presentation of endogenous antigen were upregulated and positive regulation of defense response to virus by host were downregulated (Figure 6A). Hierarchical clustering analysis of GSEA-enriched genes (Figure 6B) identified downregulated genes (shown in red) that play critical roles in B cell development and maturation, such as *CD48*, *CD74*, *CD79B*, *HLA-DPB1*, *HLA-DPA1*, *BTLA*, *BANK1*, *IRF8*, *JCHAIN*, *and FCMR*. We further analyzed the expression of five canonical B cell signature genes, including *BANK1*, *CD79A*, *CD79B*, *FCMR* and *IRF8*, based on FPKM values. It was found that the median expression of B cell signature genes in the febrile control group was consistently higher than that in the KD group (Figure 6C). These findings collectively suggest that KD iPSC-derived HSPCs display transcriptional signatures consistent with impaired B-cell developmental programs.

## 4 Discussion

### 4.1 Observations of iPSC-derived HSPC transcriptional dysregulation and corresponding peripheral immune features in KD

Accumulating clinical and basic studies have confirmed that peripheral immune dysfunction is a core pathological feature involved in the occurrence and progression of KD, and genetic susceptibility also acts as an indispensable predisposing factor driving individual differences in disease onset and severity (17–20). Consistent with previous clinical reports focusing on peripheral immune disorders in patients with KD, our clinical phenotypic data showed significant decreases in the percentage of CD4+ T cells and serum IgA levels in patients with KD compared with age-matched children with pneumonia, accompanied by a mild but statistically insignificant reduction in peripheral CD19+ B cell proportion. These observations further support the consensus that adaptive immune imbalance in peripheral circulating immune cells participates in the acute immune inflammatory response of KD.

Previous single-cell RNA-seq studies have mainly focused on mature peripheral immune cells to characterize immune alterations of KD, while upstream HSPC-related mechanisms remain poorly explored. Clinical investigation of primary HSPCs is largely restricted due to the invasive nature of bone marrow sampling. To address this limitation, we adopted an iPSC-based modeling strategy. Using paired transcriptomic analysis of iPSC-derived HSPCs from KD and febrile control groups, we observed dysregulated immune and inflammatory pathways in KD-derived HSPCs. Key altered biological features were similar to findings from public PBMC-derived HSPC datasets of KD. Notably, it remains unclear whether these HSPC-intrinsic changes initiate KD inflammation or represent secondary responses to systemic inflammatory stimulation.

KD iPSC-derived HSPCs display transcriptional signatures consistent with impaired B-cell developmental programs. This transcriptional signature at the HSPC differentiation stage could explain our clinical peripheral blood detection results: the downward trend of peripheral CD19+ B cell proportion and significantly reduced secretory IgA level in KD patients. Key genes within these sets, such as *BANK1*, *CD79A*, *CD79B*, *FCMR* and *IRF8*, are all associated with B-cell development and maturation. *BANK1* encodes a B-cell-specific scaffold protein with lupus-risk genetic variants (21). It binds PLCγ2, TRAF6 and MyD88 to mediate BCR, CD40 and TLR signaling. Functioning as a negative regulator, BANK1 inhibits B cell activation and autoantibody generation. Loss of *BANK1* expands autoreactive follicular and marginal zone B cells and aggravates autoimmune responses (22). *CD79A* (Igα) and *CD79B* (Ig*β*) encode BCR signaling heterodimer subunits essential for B-cell development and activation. Their cytoplasmic ITAMs recruit SYK via LYN phosphorylation, triggering PI3K/NF-κB cascades (23). *FCMR*, the Fc receptor for IgM, regulates bone marrow B-cell development, mature B-cell homeostasis, and marginal zone B-cell generation. Loss of *FCMR* leads to aberrant B-cell development and maturation arrest (24). *IRF8* serves as a pivotal transcription factor governing B-cell lineage specification, follicular vs. marginal zone B-cell fate decision, and germinal center formation, acting as a master regulator of B-cell maturation (25). Although our transcriptomic data reveal correlative changes in multiple key B-cell regulatory genes, direct functional evidence demonstrating their causal roles in KD-associated immune disturbances is still lacking. Our findings nevertheless provide candidate molecular targets for future mechanistic investigations into impaired B-cell development in KD.

### 4.2 Limitations of this study

This study has several notable limitations that should be acknowledged. Most importantly, this research focuses on only three patients with KD and three febrile controls, representing an extremely small sample size that substantially limits the statistical power and generalizability of our findings. Collectively, the small sample size introduce potential bias stemming from uneven gender distribution between groups, individual donor heterogeneity, and unavoidable batch effects during iPSC reprogramming and HSPC differentiation, which may confound the observed transcriptomic profiling results. In addition, all key findings in the present study are exclusively derived from transcriptomic bioinformatics analyses, without complementary *in vitro* functional experiments to validate these transcriptomic alterations. Therefore, further well-designed studies with larger sample cohorts and comprehensive functional phenotypic assays are warranted to validate and extend our preliminary observations.

## Data Availability

The datasets presented in this study can be found in online repositories. The names of the repository/repositories and accession number(s) can be found in the article/Supplementary Material.
