# Supplementary material for: Preliminary transcriptome profiling of induced pluripotent stem cell-derived hematopoietic stem and progenitor cells in Kawasaki disease
Source: Front Pediatr. 2026 Aug 19;14:1833402. doi: 10.3389/fped.2026.1833402 (PMC13533899; doi:10.3389/fped.2026.1833402)
Supplement: Supplementary file 1 [file Supplementaryfile1.docx]

**Supplemental information**

**Figure S1. Principal component analysis (PCA) of bulk RNA-seq from iPSC-derived HSPCs and public single-cell transcriptomic datasets from patients with KD, febrile controls, and healthy individuals.**

(A) PCA plot of bulk RNA-seq data from iPSC-derived HSPCs comparing patients with KD and febrile controls.
(B) PCA plot of public single-cell datasets including patients with KD, febrile controls and healthy individuals.
(C) Integrated single-cell analysis combining febrile controls, healthy controls, and pooled pre-IVIG single-cell datasets from four patients with KD.
